# Spodick’s Sign: A Case Report and Review of Literature

**DOI:** 10.7759/cureus.11606

**Published:** 2020-11-21

**Authors:** Basel Abdelazeem, Emad Kandah, Mariem Borcheni, Saed Alnaimat, Arvind Kunadi

**Affiliations:** 1 Internal Medicine, McLaren Health Care, McLaren Flint, Michigan State University, Michigan, USA; 2 Internal Medicine, Sfax Faculty of Medicine, Sfax, TUN; 3 Cardiology, McLaren Health Care, McLaren Flint, Michigan State University, Michigan, USA

**Keywords:** spodick’s sign, acute pericarditis, case report, stemi

## Abstract

Acute pericarditis is commonly diagnosed in patients who present with chest pain. Accurate diagnosis of acute pericarditis is essential because of its relative similarity to ST-elevation myocardial infarction (STEMI) in both clinical presentation and electrocardiogram (EKG) changes. Additionally, troponin elevation is occasionally seen in acute pericarditis due to myocardial involvement (myopericarditis), which makes accurate diagnosis more challenging. A 12-lead EKG remains the most useful diagnostic test in differentiating acute pericarditis from STEMI. Spodick's sign is a less recognized electrocardiographic feature of acute pericarditis and is frequently overlooked by clinicians. We present a case of a 52-year-old male who initially presented with acute onset substernal chest pain. His EKG revealed diffuse subtle ST elevation and downsloping TP segment (Spodick's sign). A coronary angiogram demonstrated normal coronaries which eliminated the possibility of coronary artery disease. In this article, we will discuss how to differentiate between acute pericarditis and myocardial infarction, with a focus on Spodick's sign, amongst other EKG findings suggestive of pericarditis.

## Introduction

The pericardium is a double-walled, thin, avascular sac that has two layers; an outer fibrous layer and an inner serous layer. The pericardial cavity normally contains about 30 to 50 ml of plasma ultra-filtrate, which acts as a lubricant to reduce friction within the prericardium, and to provide mechanical protection for the heart [1]. Acute pericarditis is an inflammatory condition of the pericardium that could be caused by infectious or noninfectious etiologies [2]. Pericarditis is classified as acute (<3 months), recurrent (repeated episodes of acute pericarditis), or chronic (>3 months) in duration [3]. Acute and recurrent pericarditis are relatively common in clinical practice. Approximately 30% of patients who develop acute pericarditis develop recurrence after the initial episode​​​​​​ [4]. Clinical presentation can vary considerably, ranging from asymptomatic to life-threatening. Patients classically present with pleuritic chest pain that is alleviated by leaning forward. On examination, pericardial friction rub may be heard. Pericarditis has four stages on electrocardiogram (EKG) based on ST and T wave changes [3]. Spodick’s sign refers to a downsloping TP segment, best visualized in lead II and lateral precordial leads [5]. It was first described by Dr. David Spodick in 1974, and is found in about 29% of patients with acute pericarditis. It represents an important clue in differentiating acute pericarditis from ST-elevation myocardial infarction (STEMI) [2-3].

## Case presentation

**Figure 1:**
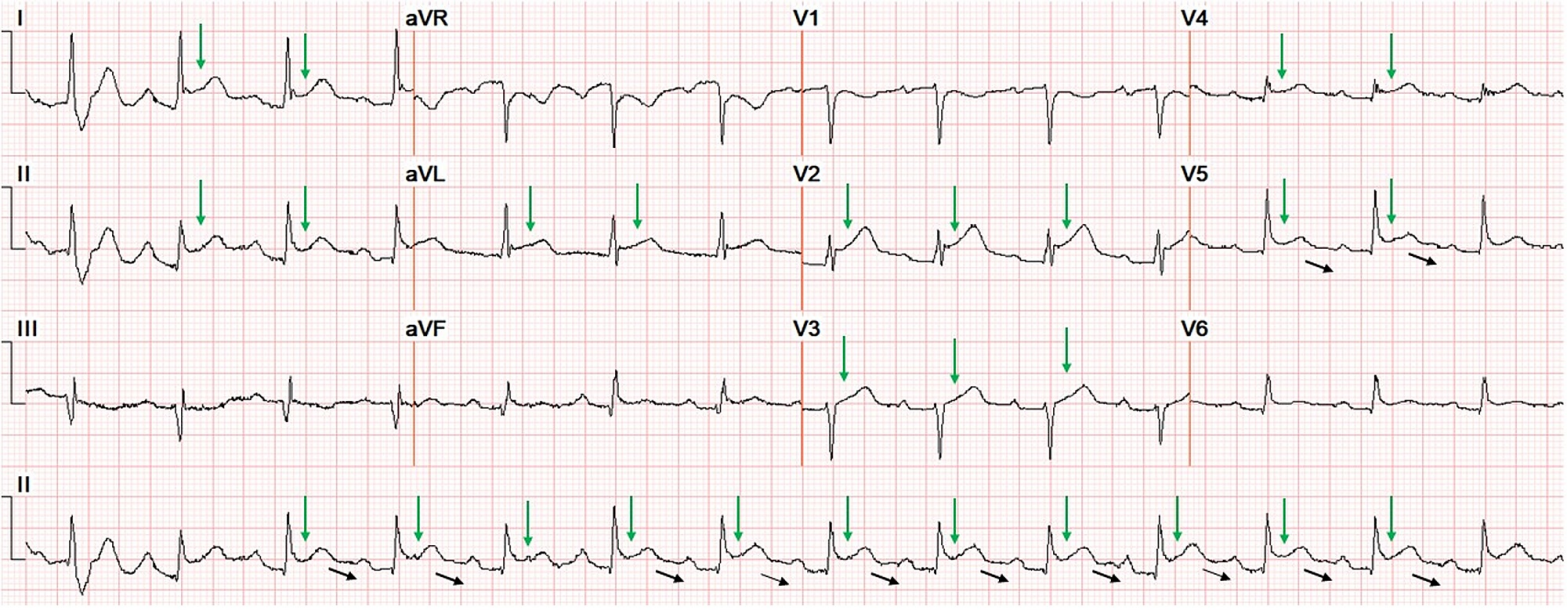
Diffuse ST elevation (green arrow) and downsloping TP segment (black arrows)

**Figure 2:**
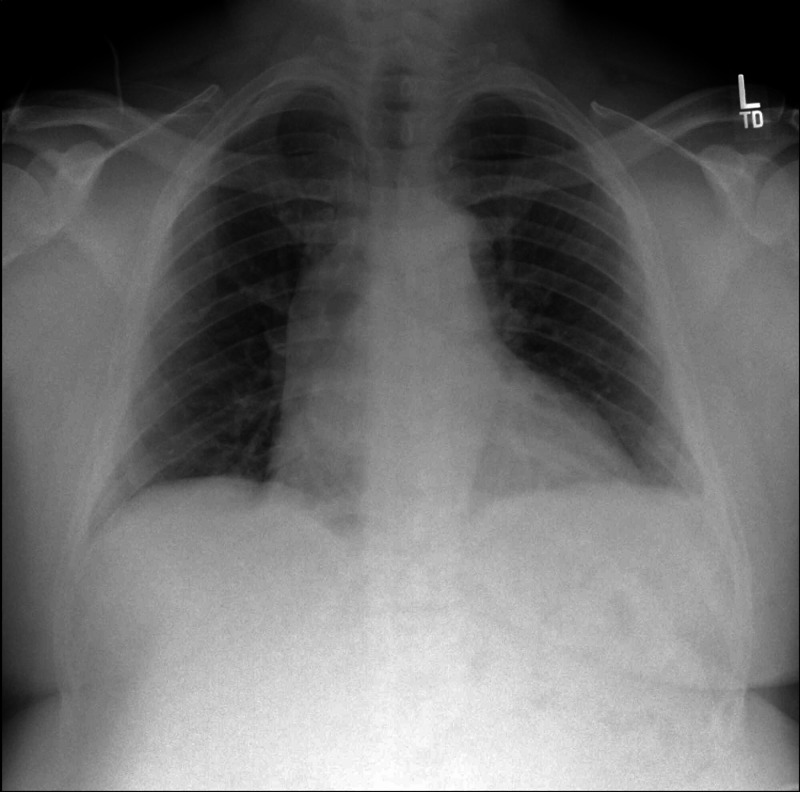
Chest X-ray showed mild cardiomegaly

A 52-year-old African American male with a past medical history of hypertension, asthma, chronic kidney disease stage-3, and obesity, presented with acute onset retrosternal chest pain for two-day duration; the pain was intermittent, mild 2/10 in severity, and worsened with exertion and inspiration. On physical exam, the patient was afebrile, with regular heart rate and rhythm. Auscultation revealed normal S1 and S2 without any cardiac murmurs or pericardial friction rub. His EKG revealed diffuse, subtle ST elevation in addition to Spodick’s sign (Figure 1). He denied any recent fever, chills, sore throat, or viral symptoms. Laboratory studies were significant for elevated erythrocyte sedimentation rate (ESR), elevated C-reactive protein, and a normal troponin-I (Table 1). Routine chest X-ray demonstrated mild cardiomegaly (Figure 2). Echocardiography revealed a left ventricular ejection fraction of 70% and a trivial pericardial effusion (Videos 1-2). Coronary angiogram revealed normal coronary arteries (Videos 3-4). The patient was started on treatment for pericarditis with renally dosed colchicine and aspirin. He reported improvement of his symptoms on subsequent outpatient follow up. 

**Table 1:** Laboratory workup WBC: white blood cells; RBC: red blood cells; BUN: blood urea nitrogen; eGFR: estimated glomerular filtration rate.

Basic Metabolic panel	Complete blood count
Glucose 127 H (70-105 mg/dL)	WBC Count 11.44 H(4.50-11.00 X 10*3/uL)
BUN 21(7-22 mg/dL)	RBC Count 3.65 L(4.70-6.10 X 10*6/uL)
Creatinine 2.30 H(0.50-1.50 mg/dL)	Hemoglobin 10.6 L(13.5-17.7 g/dL)
eGFR African American 36 L(60-200 mL/min)	Hemoglobin 10.6 L(13.5-17.7 g/dL)
BUN/Creatinine Ratio 9.1 L(12.0-20.0)	Platelet Count 271(140-440 X 10*3/uL)
Calcium 8.5(8.5-10.5 mg/dL)	Neutrophils, Automated 69.5( %)
Sodium 138(134-145 mM/L)	Lymphocytes, Automated 15.0( %)
Potassium 3.1 L(3.5-5.1 mM/L)	
Chloride 98(98-112 mM/L)	
Carbon Dioxide 27(24-30 mM/L)	
Anion Gap 13.0(6.0-14.0 mM/L)	
C Reactive Protein 19.1 H(0.0-0.9 mg/dL)	Troponin-I 0.03(0.00-0.04 ng/mL)
Sedimentation Rate Westergren 60 H(0-20 mm/Hr)	

**Video 1:** Echocardiogram revealed a left ventricular ejection fraction of 70% and a trivial pericardial effusion

**Video 2:** Echocardiogram revealed a left ventricular ejection fraction of 70% and a trivial pericardial effusion

**Video 3:** Coronary angiogram showed normal coronary arteries

**Video 4:** Coronary angiogram showed normal coronary arteries

## Discussion

Acute pericarditis is diagnosed in about 0.1% of hospitalized patients and 5% of patients presenting to the emergency department with chest pain after ischemic causes were ruled out [6]. The majority of diagnosed cases (up to 75%) are idiopathic [7]. Infectious etiologies include viral infections (e.g. Coxsackie virus, which is the most common infectious etiology); tuberculosis, which is a common cause of pericarditis in developing countries [3]; and other bacterial or fungal infections. Post myocardial infarction pericarditis can occur early (within two to four days) in up to 25% of patients, or late as Dressler syndrome, in which autoimmune pericarditis occurs weeks to months after myocardial infarction [8]. Neoplastic etiologies also should be considered and include primary tumor of the pericardium such as mesothelioma and sarcoma, metastatic spread, or direct invasion from a nearby lung/breast cancer or lymphoma [9]. Other causes to consider may include metabolic disorders (such as uremia, hypothyroidism), autoimmune connective tissue diseases (such as rheumatic fever, systemic lupus erythematosus, and rheumatoid arthritis [10]). Additionally, pericarditis can be traumatic secondary to cardiothoracic surgeries or chest trauma [11].

Most cases present with pleuritic chest pain, substernal or left-sided, severe, sharp that increases with inspiration and improves with leaning forward [12]. Occasionally, patients may report a prodrome of fever, malaise, and myalgia. On physical examination, patients may have tachycardia and pericardial friction rub (due to friction between the two inflamed pericardial layers) [13].

EKG plays a major role in diagnosing acute pericarditis. However, EKG features can be seen in no more than 60% of the cases. Although the pericardial sac itself lacks electrical activity, inflammation of the pericardium can disrupt the action potential in the adjacent myocardium (epicardium), leading to characteristic ST segment elevation on EKG. A local insult to myocardial cells can result in distortion of the shape of action potential, causing a voltage gradient (current of injury) between injured myocardial cells and surrounding unaffected cells. This current of injury is reflected in ST segments on vectors pointing towards a lesion, leading to ST segments elevation or depression with variable morphologies. Generally, ST segment elevation occurs in transmural or epicardial injury, whereas ST segment depression occurs if subendocardial insult is present. Due to the diffuse nature of pericardial inflammation, the current of injury does not correspond to a specific coronary territory as seen in cases of STEMI. Involvement of the atria is responsible for PR segment depression and is quite specific for pericarditis [14].

Repolarization abnormalities of acute pericarditis undergo different chronological phases, which are summarized in Table 2. The suggestive findings of acute pericarditis are summarized in Table 3. These changes may not be present in every patient with acute pericarditis. Important electrocardiographic clues in differentiating acute pericarditis from myocardial infarction are summarized in Table 4 [15-18].

**Table 2:** EKG findings consistent with pericarditis in the different chronological stages

Stages	stage I	stage II	stage III	stage IV
Time frame	Hours to several days	A few days to weeks	Several weeks	Months
EKG findings	PR-segment depression, except in aVR	Normalization of ST and PR segments	T-wave inversion persists with isoelectric ST- segments	Gradual resolution of T-wave inversion
	PR segment elevation in lead aVR	T waves progressively flatten and invert.		Diffuse ST-segment depression and T-wave inversion may persist up to 3 months.
	Diffuse concave-upward ST-segment elevation with concordant (upright) T waves			
	Absence of reciprocal ST-segment changes			
	Leads aVR and V1 may have ST-segment depression			

**Table 3:** EKG findings suggestive of acute pericarditis

EKG findings suggestive of acute pericarditis
ST-elevation is less than 5 mm
ST-segment concavity
More extensive lead involvement
Less prominent reciprocal ST-segment depression
PR-segment elevation in aVR, with reciprocal PR-segment depression in other leads
The absence of abnormal Q-waves
Variability in the time of T-wave inversion occurrence following ST-segment elevation
The lack of QRS widening and QT interval shortening in leads with ST-elevation

**Table 4:** Comparison of EKG findings in acute pericarditis and myocardial infarction

Acute pericarditis	Myocardial infarction
ST-segment elevation usually "concave" upward (bulge downward)	ST-segment elevation usually "convex" downward (bulge upward) during acute injury
Diffuse ST elevation	ST-elevation related to location of ischemia
No Q waves or reciprocal changes are seen	Q waves often appear
PR-segment depression often occurs	No PR-segment depression

Spodick's sign, first described in 1974, is an important clue that may assist clinicians in differentiating between STEMI and pericarditis. Spodick's sign is a downsloping of the TP segment mainly seen in lead II and lateral precordial leads [5].

According to the European Society of Cardiology, acute pericarditis requires the presence of two or more of the following: pericarditic chest pain, pericardial friction rub, new widespread ST-segment elevation or PR depression, or new or worsening pericardial effusion [9]. Our patient presented with chest pain and diffuse ST segment elevation on EKG, and echocardiography revealed a trivial pericardial effusion meeting the diagnostic criteria for acute pericarditis.

In a recently published retrospective study that included 165 patients with STEMI and 42 patients with pericarditis, Spodick's sign was noticed in 5% of patients with STEMI (95% CI 3%-10%) and 29% of patients with pericarditis (95% CI 16%-45%). No single EKG sign was pathognomonic for STEMI or pericarditis. Up to 12% of patients with STEMI had PR segment depression. This data was collected from a single site and has a small number of pericarditis patients, limiting the accuracy of results and generalizability of findings. Further studies are needed to validate these results [16].

Echocardiography is helpful in assessing cardiac function and presence of pericardial effusion [19]. ESR and C-reactive protein are markers of inflammation and are frequently elevated in patients with acute pericarditis. Troponin elevation is seen in patients with myocardial involvement (myopericarditis). The most common complications of acute pericarditis are pericardial effusion and cardiac tamponade. Up to 60% to 75% of patients with acute pericarditis may develop pericardial effusion [7,9,13,18].

## Conclusions

EKG remains the most useful diagnostic test in differentiating acute pericarditis from STEMI. Among several EKG findings that may suggest pericarditis, Spodick's sign is an important diagnostic clue that is commonly overlooked by clinicians. However, Spodick’s sign is not pathognomonic for acute pericarditis.
